# Implementing clinical ethics committees as a complex intervention: presentation of a feasibility study in community care

**DOI:** 10.1186/s12910-020-00522-1

**Published:** 2020-09-01

**Authors:** Morten Magelssen, Heidi Karlsen, Reidar Pedersen, Lisbeth Thoresen

**Affiliations:** 1 Centre for Medical Ethics, Institute of Health and Society, University of Oslo, Pb. 1130 Blindern, N-0318 Oslo, Norway; 2 Department of Interdisciplinary Health Sciences, Institute of Health and Society, University of Oslo, Oslo, Norway

**Keywords:** Clinical ethics, Clinical ethics committee, Clinical ethics support services, Complex intervention, Ethics in community care, Healthcare ethics

## Abstract

**Background:**

How should clinical ethics support services such as clinical ethics committees (CECs) be implemented and evaluated? We argue that both the CEC itself and the *implementation* of the CEC should be considered as ‘complex interventions’.

**Main text:**

We present a research project involving the implementation of CECs in community care in four Norwegian municipalities. We show that when both the CEC and its implementation are considered as complex interventions, important consequences follow – both for implementation and the study thereof. Emphasizing four such sets of consequences, we argue, first, that the complexity of the intervention necessitates small-scale testing before larger-scale implementation and testing is attempted; second, that it is necessary to theorize the intervention in sufficient depth; third, that the identification of casual connections charted in so-called logic models allows the identification of factors that are vital for the intervention to succeed and which must therefore be studied; fourth, that an important part of a feasibility study must be to identify and chart as many as possible of the causally important contextual factors.

**Conclusion:**

The conceptualization of the implementation of a CEC as a complex intervention shapes the intervention and the way evaluation research should be performed, in several significant ways. We recommend that researchers consider whether a complex intervention approach is called for when studying CESS implementation and impact.

## Background

Clinical ethics support services (CESS) aid in the handling of ethical problems that arise in clinical practice [1, 2]. A common form of CESS is the clinical ethics committee (CEC). CECs have traditionally had three main tasks: Deliberation on clinical-ethical problems that either involve particular patients or more general ethical issues; education of clinical staff in topics pertaining to clinical ethics; and contributions to the development of institutional guidelines [3].

In these ways – and also through other activities [3] – CECs contribute to improved skills, knowledge and awareness of clinical ethics and better handling of ethically challenging situations that arise in practice [4]. However, establishing a successful CEC is no straightforward matter. Through working with the Norwegian CECs, our experience is that it takes several years to build up competence and experience among CEC members, to overcome barriers, and to establish the CEC firmly as a structure which has a good standing among staff in the services and which staff make use of [5–7]. Furthermore, successful implementation is not done once and for all; it takes effort to maintain a well-functioning CEC. In this paper we ask, how should clinical ethics committees (CECs) be implemented and evaluated? Our answer is that both the CEC itself and the *implementation* of the CEC should be considered as “complex interventions”. We argue by way of example, through presenting (in some depth) the plans for a research project involving the implementation and evaluation of four CECs. No empirical findings from the project will be presented here; these will be presented in a set of articles to come.

### The implementation of a clinical ethics committee as a complex intervention

Our experiences have led us to conceptualize the CEC itself as a *complex intervention*, as first proposed by Schildmann et al. [8, 9]. We suggest that in addition, the implementation of a CEC within a healthcare organization should also be considered as a complex intervention. This has consequences both for how we theorize the interaction between the CEC and the healthcare services and professionals that it is to serve [8], and for how the CEC should be implemented and evaluated.

Although CESS have become widespread in somatic hospitals in many Western countries, such services have typically not been established in community/primary/municipal care services. In this article we describe a project where researchers from the Centre for Medical Ethics (CME) at the University of Oslo will aid four municipalities in establishing CECs for their health and care services. The establishment of new CECs can be seen as a complex intervention where the complexity is due both to the multiple components and actors that will interact to effect change, the multiplicity of organisational levels involved, the need for flexibility in tailoring the intervention and the implementation support, and the potential range of outcomes [9–11]. The relationship between components, actors and outcomes can helpfully be visualized in a so-called ‘logic model’ (also known as ‘conceptual framework’) [10]. In what follows, special emphasis will be given to the conceptualization of CEC as a complex intervention and what this entails both for the establishment of CECs and for the research design. Specifically, being conscious of the complexity, uncertainty and local variation involved in the design and implementation of the CECs itself, the study is framed as a feasibility study, as we recognize that factors concerning feasibility, process and relevant outcomes must be studied ahead of any larger-scale outcomes studies such as randomized controlled trials (RCTs) or mixed method studies.

### The Norwegian context

In Norway the municipality has a central place in organizing and providing primary health and care services to inhabitants. As of 2018 there were 422 municipalities, ranging from the very small (< 5000 inhabitants) to the large (16 municipalities have > 50,000 inhabitants). There are clear signals from the national political level that municipalities have a responsibility to promote ethics reflection and knowledge of ethics among staff [12].

Through a national program which ran from 2007 to 2015, 243 municipalities were aided in developing local initiatives to promote ethics reflection and knowledge. The most prevalent initiative was so-called ethics reflection groups (ERGs) [13]. Here, professionals within a department meet to discuss concrete cases/situations that they experience as challenging. However, only approximately a quarter of municipalities had active ethics activities as of 2015 [13]. What is more, the activities only involved parts of community care, mainly nursing homes and home-based care [14–17]. Yet, ethics support is likely to be just as important in other parts of municipal health and care services, such as local public health centres, sheltered housing, school health services, substance abuse and mental health services, and for general practitioners. It is this set of services that we refer to throughout as ‘primary’, ‘community’ or ‘municipal’ health and care services; we use these three designations interchangeably.

ERGs’ main advantage is proximity to the services, employees and clinical work. Worthwhile as they are, ERGs cannot respond to all the needs of the services when it comes to ethics. Therefore, it might be thought helpful to supplement them with a CEC for the entire health and care sector in the municipality. The CEC is a multidisciplinary group with typically 8–12 members, often including one or more user/lay representatives. CECs assist the services in discussing specific clinical-ethical problems from practice that professionals, patients or next of kin ask for help with. Unlike the typical ERG, a CEC aspires to include all stakeholders in the deliberations and routinely produces written reports. It is a more robust structure with greater ethics competence and a more formal position in the municipality. The purpose of CEC deliberations is to promote reflection on important issues from practice and give advice when requested. CECs are sometimes also tasked with overseeing and coordinating the totality of the municipality’s efforts within ethics in the health and care sector. This includes hosting seminars on ethics-related topics.

The CME receives a yearly grant from the Ministry of Health and Care Services to coordinate work in the CECs and improve competence. In Norway, all hospital trusts are obligated to have a CEC, and as of 2018 there were 38 hospital CECs [18]. In the Norwegian municipal sector, however, CECs are rather novel structures; as of 2015 there were only nine municipal CECs [19]. An evaluation study reports that the municipal CECs are perceived as significant contributors yet fragile structures, and that there is a major potential for improvement in their work and conditions. Expertise and experience in clinical ethics, case deliberation and committee leadership are probably key success factors; so is sufficient support from municipal leadership [19].

Internationally, there are few reports detailing the prevalence, process of establishment, activities and evaluation of clinical ethics committees (CEC) in community care [20]. In several countries there are CECs that serve nursing homes, yet such committees apparently seldom cover other community health and care services. Outside of Norway we are not aware of CECs that aim to cover a community or municipality’s entirety of primary health and care services. We were unable to locate published research about the establishment of such CECs outside of Norway.

The CEC which covers a large range of community health and care services is, then, a relatively novel structure, and the research project will be novel also in an international context. The project will provide knowledge about process and outcomes that will be sensitive to the specific Norwegian context, yet it can also inform and inspire similar efforts internationally.

## Main text

In this section, the proposed research project’s design will first be detailed. Then, we will discuss and highlight the ways in which the ‘complex intervention’ framework has decisive impact on aspects of the study design.

### Study design

The research project is a feasibility and process evaluation study which will study several aspects of the establishment, implementation and early operation of CECs in four Norwegian municipalities. The project commenced in January 2018 and will continue for 2.5 years. Data collected will be predominantly qualitative, yet also quantitative. Through multiple research methods detailed below we aim to gain in-depth and contextual knowledge about the establishment of municipal CECs. Results may inform the planning and execution of a future larger-scale implementation study with, for instance, cluster RCT or stepped wedge design.

### Aim and research questions

The overarching aim of the project is to implement, describe and evaluate CECs as a structure for working with ethics in community health and care services. We ask the following three main research questions (RQs):

- RQ1: Do the CECs strengthen professionals’ ethics competence and handling of ethical problems, and if so, how?
- RQ2: Which factors promote or inhibit the successful establishment and implementation of a CEC?
- RQ3: Is the CEC capable of furthering the involvement of patients and next of kin in municipal health and care services?

### Evaluation criteria

Core evaluation criteria are presented in Table 1, grouped according to three central domains in evaluation: Structure, process and outcome [21, 22]. The criteria were partly inspired by the American Society for Bioethics and Humanities’ (ASBH) standards for ethics consultation [23].

**Table 1:** Core evaluation criteria

*Structure*	
1. CEC is established within first 6 months and operative at end of study period 2. The CEC receives adequate support from municipality leadership: formal support, funding, time for CEC members to participate 3. Key groups are represented as CEC members: doctor, nurse, professional actively involved in clinical work, layperson 4. All stakeholders (professionals, managers, patients, next of kin) have formal and practical access to the CEC	
*Process*	
5. At least 6 CEC meetings yearly in study period 6. At least 3 visits to services in study period 7. Patient and/or next of kin have been invited to participate in at least two of the case discussions 8. All case deliberations from final 12 months have been documented in writing 9. Routines for handling ethics cases have been established 10. Patient or surrogate consent is sought whenever their case is discussed in the CEC	
*Outcome*	
11. Having received and deliberated on at least 2 ethics cases from the services within final 12 months 12. Having held at least one ethics seminar for professionals within final 12 months 13. Having made a plan within the first 12 months for making the CEC known in the services, and having executed the plan within the study period 14. CEC case deliberation making a difference for practice and for stakeholders, according to stakeholders themselves (professionals, managers, patients, next of kin) In addition, other potentially important outcomes should be defined in the course of the project in cooperation with stakeholders.	

### Participating municipalities

Throughout the planning phase we came into contact with five municipalities who were already interested in establishing CECs. One municipality withdrew interest, but the other four agreed to participate in the project and have signed a contract detailing the terms of cooperation. Varying in size from just over 10,000 to over 80,000 inhabitants, the four municipalities also vary concerning the organization of the services and the preparatory work for establishing a CEC that was performed prior to the commencement of the project. The municipalities will have the opportunity to influence planning, constitution, organization and implementation of the CEC to adapt the intervention to local circumstances and needs. A key tenet of a complex intervention framework is that such local adaptations are inevitable. They might be positive, in that a better ‘contextual fit’ is thereby achieved, or negative, in that the desired causal effects of the intervention might be attenuated. The municipalities were asked to appoint two key persons each, to receive training and follow-up from CME (see below) and become leader and secretary of the CEC respectively.

### Description of the intervention

Participating municipalities are committed to establishing CECs within the first 6 months and to provide the necessary resources and support. The committees are recommended to have 8–12 members recruited mainly among municipal employees. Broad, multidisciplinary representation from the services that the committee will assist is required, and at least one layperson should be included. Although the CEC’s organization will have to be adapted to the municipality’s needs, we recommend that the committee in time will become the central coordinator of the entirety of the municipality’s ethics activities in the health and care sector.

The CECs will be urged to use a discourse ethics model (the CME 6-step model [24]) to structure the ethics deliberations and written case reports. CECs should also invite all relevant stakeholders to participate in deliberations when relevant and feasible. CECs will be encouraged to develop routines and forms, such as standardized written information about the CEC. The data collection will last 2.5 years, yet the CECs are expected to be operational and self-sustaining beyond that time frame.

The key persons will be educated in ethics through participation in CME’s three master-level courses in healthcare ethics (each at 5 ECTS and each comprising five full days of teaching plus readings), focusing on 1) ethical theory and argumentation, 2) clinical ethics, and 3) facilitation of ethics deliberation and leadership of ethics activities, respectively. This is intended to equip the key persons with the necessary skills for running the CEC, including handling ethics cases referred to the CEC from the services.

**Fig. 1:**
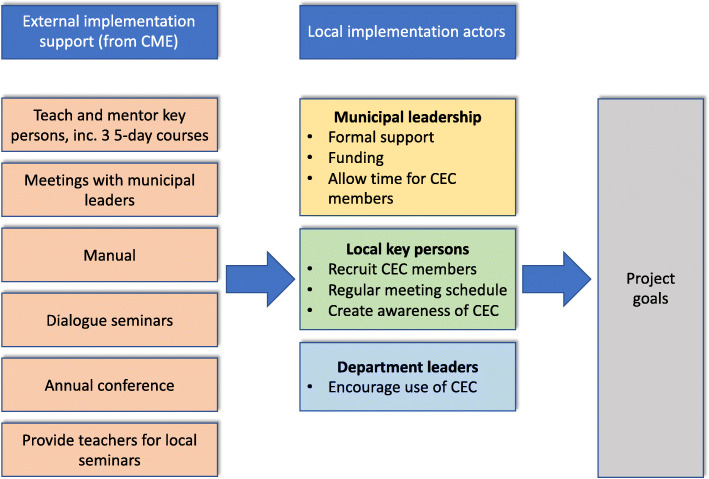
Logic model for the implementation of municipal CECs

**Fig. 2:**
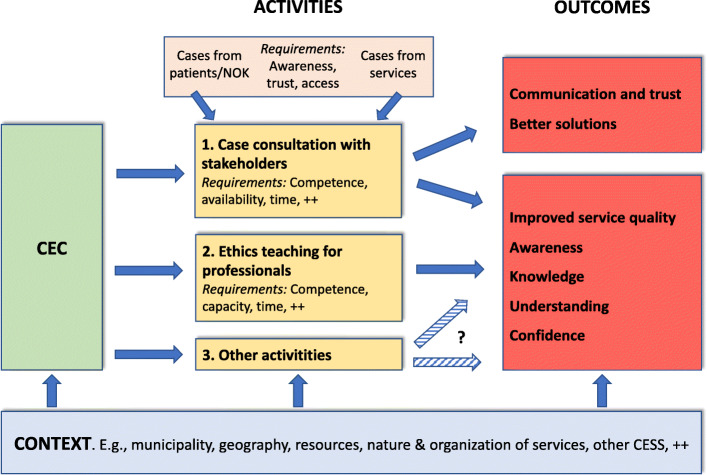
Logic model for the operation of the CEC in the municipality, highlighting some central features of the context, the CEC and its activities, desired/hypothesized outcomes, and likely requirements. Arrows indicate likely influences and causal links

The project group at the CME will have an important role in supporting the CECs and especially the key persons. In addition to the formal training described above, the CME aids the CECs and the key persons in the following six ways: 1) Through *participating in meetings with municipal leaders* in the planning phase if requested. 2) Through *dialogue seminars* where all key persons across the participating municipalities take part, once or twice yearly. Here, key persons share experiences and discuss challenges for their work in the CECs with input from project members. 3) Through yearly *2-day conferences* where all members of all municipal CECs are invited. 4) Through CME’s *written manual* for municipal CECs. 5) Through *visits* to the CECs or *participation in ethics seminars* for employees when requested. 6) Through ad hoc *guidance* when needed. The dialogue seminars are thought to be especially important, in that they allow the interchange of information («feedback loops») which might both benefit the key persons and their CECs and lead to adjustments of the research project. As participants are likely to have different needs, it will be important that the CME offers close follow-up, guidance and practical assistance tailored to the needs of the municipality and key persons. Key project inputs, components and causal relationships are illustrated in the logic models depicted in Fig. 1 and Fig. 2.

### Data collection

In order to assess different aspects of the intervention, a range of data sources are employed (Table 2).

**Table 2:** Data sources in the project and the main topics they address

Qualitative data		
	*Data source*	*Main topics*
1	Three focus group interviews with key persons, in addition to audio recordings and notes from all dialogue seminars	Key persons’ experiences with work in the CECs, experienced challenges, and perceived impact of the training received
2	One focus group interview with each of the participating CECs in full, towards the end of the study period	Experiences and challenges with work in the CEC, including case deliberation. Perceived impact on services, municipality and CEC members
3	Individual interviews with the head of each municipality’s health and care sector	Perceived impact on services and municipality. Municipal support for the CEC
4	Individual interviews (up to 20) with professionals who have been involved in a case discussed in the CEC	Practical consequences of CEC involvement. Experiences with taking part in CEC deliberations
5	Individual interviews (up to 15) with patients and next of kin who have been involved in a CEC deliberation	Practical consequences of CEC involvement. Experiences with taking part in CEC deliberations
6	CEC deliberation reports (anonymized)	Nature of ethical issues. Characteristics of the CECs’ ethical reasoning
7	Observation of CEC deliberations (1–2 per CEC)	Deliberation process. Involvement of stakeholders
8	CECs’ yearly reports	CEC activities such as seminars, other outreach, number of attendees, services involved. CEC members
9	CEC deliberation reports (anonymized) and committee’s self-evaluation form for each case	Quantitative data about CEC cases

### User involvement

In the project, user involvement at several levels is key. Three different groups of users are involved:

1. *Patients, next of kin and professionals who contact the CEC and/or participate in case deliberations.* Ethics deliberation in the CEC is an opportunity for stakeholders to present their viewpoints, contributing to a fuller picture of the ethical challenge at hand. Norwegian CECs attempt to include stakeholders in cases where they are involved, and the strengthening of patient autonomy and shared decision-making in the services is an underlying ethical commitment for the CEC [4]. Stakeholder perspectives and feedback must be used constructively by the CECs and by the project for quality improvement including refinement of routines in connection with case deliberations.
2. The role of the *two key persons in each CEC* was introduced above. As representatives of their CECs they will be important partners throughout the project, from planning to post-project evaluation, and their experiences should inform both the intervention and the evaluation. For instance, they will provide feedback on drafts for data collection forms, and their experienced challenges will be brought up in dialogue seminars.
3. *Participating municipalities* are organized differently and have different needs. Therefore, it is necessary for the project to tailor the CEC’s characteristics to local needs, in cooperation with municipal management and the appointed key persons. For instance, municipalities are likely to have different views on which specific services should be served by the CEC.

### Research ethics

The research will involve patients and next of kin, but only patients who are competent to consent will be invited. Next of kin will be particularly important to include in situations in which the patient is unable to participate. CEC case deliberation presupposes informed consent from involved parties unless the case is sufficiently anonymized. Whenever person-identifying information is registered, participants to the CEC meetings will consent to participate in the research.

### Conceptualizing as a complex intervention – and the difference that it makes

In this section we explore the question of what difference the conceptualization of the establishment of the CEC as a *complex intervention* ought to make for the project. The evaluation of complex interventions in healthcare has increasingly been recognized as a distinct research paradigm [25]. The degree of complexity depends on factors such as the number of target groups, the number and complexity of interacting components, the difficulty of the implementation and the degree of flexibility required [8, 10]. As has been argued, in all these respects the present project is highly complex.

The conceptualization of the implementation of CECs as a complex intervention has four main consequences for the project that we will highlight. First, acknowledging the multiple dimensions of complexity means that it is necessary to perform small-scale testing of the intervention before it is tried out on a larger scale in a controlled study. Accordingly, the present project has been designed as a feasibility study, examining the conditions for the intervention to succeed. It is also a pilot study in that it tests the intervention on a small scale to explore whether it is fit to be applied widely. At this stage it is helpful to gather in-depth qualitative data that provide an elaborate understanding of the intervention, combined with basic quantitative measures [10]. One cannot take for granted that a complex intervention will in fact be used, or that it will operate fully as intended. Thus, a thorough evaluation is warranted, studying both implementation outcomes, outcomes for services and professionals, and outcomes for patients/relatives [26]. For instance, if unsuccessful on the first two levels, there will be no positive outcomes of the intervention for patients/relatives. This although there might not necessarily be anything wrong with the intervention itself. It is also important to distinguish the concrete intervention (i.e., the CEC) from the intervention constituted by the implementation [27]. Again, if positive outcomes are lacking this might then not necessarily indicate a deficiency in the intervention itself; it could also be due to deficiencies in the implementation support.

The dimensions of complexity also make the construction of sound larger-scale studies particularly challenging. For instance, relevant outcomes might be on different levels: individual patient, whole ward, institution, or whole municipality. It then becomes a question of what would constitute relevant comparisons in a controlled study. An RCT comparing CEC implementation using other municipalities as the control group, might be ill-suited to detect relevant outcomes on the clinical level, and vice versa. Such challenges in conducting large-scale studies make pilot studies such as the present all the more necessary.

Second, it is necessary to theorize the intervention in sufficient depth, emphasizing what exactly it consists in, its justifications and aims, and how it is used in practice. As Schildmann et al. argue, understanding the clinical ethics support intervention – and here a complex intervention perspective is helpful – is necessary in order to devise appropriate means of evaluating it [9]. We must understand not only the CEC’s outcomes, but also its mechanisms of impact; building on preconceptions from the outset [9], this understanding must then be adjusted in light of the findings. How does the intervention effect change? [10] This requires an in-depth understanding of what CECs actually do. In the project we examine this with a number of methods that complement each other, from observation of CEC deliberation and interviews with stakeholders about experiences and perceived consequences to CEC members’ self-assessment of each deliberation and independent evaluation of the ethical reasoning in the case minutes. The intention is both to identify important outcomes of the CEC, and how the outcomes are created and what factors facilitate or attenuate them. The key causal connections can be illustrated in a logic model such as in Fig. 2. The project is likely to yield knowledge of causal connections that can lead to a revision of the logic model at the end of the project. It is also important to see the connections between the CECs’ aims and the outcomes that are relevant to study. For instance, in a discourse ethics framework [28], a CEC having good deliberations where stakeholders are able to participate would be a main goal in itself, arguably one that is more important than stakeholder satisfaction itself. If the former aim is taken as more important, efforts should be made to investigate whether and to what extent this aim is in fact met – for instance through observation of case deliberations and analysis of written case reports.

Third, the identification of casual connections charted in the logic model also allows the identification of factors that are particularly vital for the intervention to succeed. In our project, one of several such factors is for management to provide sufficient time for CEC members to participate in meetings and attend education. Another is the way that stakeholders are welcomed and included in the CEC’s deliberation. As these are crucial factors for the CEC to succeed, the feasibility study should give special emphasis to these factors and assess them with appropriate (and perhaps multiple) methods. As further examples of this, local key persons should be asked about the extent and the relative significance of the six different domains of external implementation support they have received (Fig. 1). They should also be asked about whether the other local implementation actors (Fig. 1) have in fact done what was expected of them, and what their impact has been. Work on the logic model in Fig. 1 led us to realize that we should also interview leaders of the municipal health and care services, as these would shed light on what kind of resources and formal support the CECs have received, in addition to what role and impact the municipal top leadership judges the CEC to have had. Furthermore, as the CECs depend on cases received from next of kin/patients and staff, it should be evaluated whether the hypothesized prerequisites – e.g., awareness of- and access to the CEC – are in place (Fig. 2). As a final example, Fig. 2 indicates important potential outcomes, but how are these best assessed? If the project is expected to contribute to improved service quality, then both interviews with staff, mid-level managers and more objective measurements might be warranted. In general, as Schildmann et al. argue, understanding the clinical ethics support intervention – and here a complex intervention perspective is helpful – is necessary in order to devise appropriate means of evaluating it [9].

Fourth, a complex intervention framework involves acknowledging that what might work well at one time and place might work less well or not at all at another. The project group must be aware of differences in context (e.g., municipality size and resources, pre-existing and potentially competing ethics services, professionals’ attitude to the CEC) and how these might potentially alter the intervention and/or outcomes – and might ultimately cause the intervention to fail or succeed. An important part of the feasibility study must be to identify and chart as many as possible of the causally important contextual factors. This will enable a more solid grounding for assessments of whether the CEC’s success or failure was due to, e.g., design flaws, unhelpful local adaptations, or difficult local conditions for work in the CEC. It is also fundamental when assessing outcomes to understand that the intervention is not a standard ‘package’, but might come in degrees. In a complex intervention framework, one must assess the intervention’s fidelity (to what extent it conforms to what was recommended/planned), adaptations (the local adaptations that have been made and the impact of these), dose (e.g., how active the CEC has been, including how often it has been utilized by professionals, patients and next of kin) and reach (e.g., how many professionals have been reached by seminars and what parts of the services have had access to the CEC’s services).

## Conclusion

We have described a project conceptualized as a complex intervention project, in which the implementation of CECs in four Norwegian municipalities will be studied. In sum, a complex intervention framework involves awareness that implementation takes place in a specific context and interacts with its surroundings in a myriad of ways, not all of which can be predicted. In contrast, more traditional evaluation research on CECs has typically been more narrow, studying outcomes such as stakeholder views and appreciation [4]. In doing so these studies are likely to have missed knowledge which would be helpful for the implementation of new CECs. How can management facilitate (or hinder) the successful implementation of a CEC? What kind of training is most helpful for new CEC members? How should CECs involve stakeholders in deliberations? The present project aspires to shed light on these and further important questions in ways that traditional evaluation research does not. Ultimate aims of the project are an understanding of what consequences the establishment of a municipal CEC might have for the services, the professionals, patients and next of kin; and an in-depth understanding of what it takes for the implementation of a municipal CEC to succeed. The latter will inform the development of a new implementation support package with the resources that the future municipalities interested in establishing a CEC will require. We recommend that researchers consider whether a complex intervention approach is called for when studying CESS implementation and impact.

## Data Availability

All data are presented in the article.

## Abbreviations

CEC: Clinical ethics committee
CESS: Clinical ethics support services
CME: Centre for Medical Ethics
ECTS: European Credit Transfer and Accumulation System
ERG: Ethics reflection group
RCT: Randomized controlled trial
