# Correction to: Implementing clinical ethics committees as a complex intervention: presentation of a feasibility study in community care

**DOI:** 10.1186/s12910-020-00534-x

**Published:** 2020-09-30

**Authors:** Morten Magelssen, Heidi Karlsen, Reidar Pedersen, Lisbeth Thoresen

**Affiliations:** 1 Centre for Medical Ethics, Institute of Health and Society, University of Oslo, Pb. 1130 Blindern, N-0318 Oslo, Norway; 2 Department of Interdisciplinary Health Sciences, Institute of Health and Society, University of Oslo, Oslo, Norway

**Correction to: BMC Med Ethics 2020, 21: 82**

**https://doi.org/10.1186/s12910-020-00522-1**

It was highlighted that the original article [1] contained an error in Table 2. This Correction article shows the correct Table 2. The original article has been updated.

**Table 2:** Data sources in the project and the main topics they address

**Qualitative data**		
	*Data source*	*Main topics*
1	Three focus group interviews with key persons, in addition to audio recordings and notes from all dialogue seminars	Key persons’ experiences with work in the CECs, experienced challenges, and perceived impact of the training received
2	One focus group interview with each of the participating CECs in full, towards the end of the study period	Experiences and challenges with work in the CEC, including case deliberation. Perceived impact on services, municipality and CEC members
3	Individual interviews with the head of each municipality’s health and care sector	Perceived impact on services and municipality. Municipal support for the CEC
4	Individual interviews (up to 20) with professionals who have been involved in a case discussed in the CEC	Practical consequences of CEC involvement. Experiences with taking part in CEC deliberations
5	Individual interviews (up to 15) with patients and next of kin who have been involved in a CEC deliberation	Practical consequences of CEC involvement. Experiences with taking part in CEC deliberations
6	CEC deliberation reports (anonymized)	Nature of ethical issues. Characteristics of the CECs’ ethical reasoning
7	Observation of CEC deliberations (1-2 per CEC)	Deliberation process. Involvement of stakeholders
**Quantitative data**		
	*Data source*	*Main topics*
8	CECs’ yearly reports	CEC activities such as seminars, other outreach, number of attendees, services involved. CEC members
9	CEC deliberation reports (anonymized) and committee’s self-evaluation form for each case	Quantitative data about CEC cases
